# A Special Role for Lawyers in a Social Norm Change Movement: From Tobacco Control to Childhood Obesity Prevention

**Published:** 2009-06-15

**Authors:** Samantha Graff, Jacob Ackerman

**Affiliations:** Public Health Law and Policy; Public Health Law and Policy, Oakland, California

## Abstract

The Robert Wood Johnson Foundation (RWJF) has committed $500 million to reverse the childhood obesity epidemic by 2015. To accomplish this ambitious goal, RWJF and its partners have developed a movement to tackle childhood obesity as a societal problem, calling for population-based solutions. The movement is borrowing from the "social norm change" approach that has yielded tremendous public health gains in tobacco control. The goal of a social norm change movement is to influence behavior indirectly by creating a social environment and legal climate in which harmful products and conduct become less desirable, acceptable, and attainable. This article explains the social norm change approach that has driven the highly effective tobacco control movement in California, highlights a unique role that lawyers have played in this approach, and describes how lawyers are preparing to play a similar part in the movement to prevent childhood obesity.

## Introduction

Policy makers and advocates hold up the California Tobacco Control Program (CTCP) as a model public health campaign. In November 1988, California voters approved Proposition 99, the Tobacco Tax and Health Protection Act. This landmark legislation imposed a cigarette tax of 25 cents per pack, established the CTCP in the state health department, and earmarked 20% of the new revenues for state and local programs aimed at reducing tobacco use (1).

In the ensuing 20 years, California has emerged as an international leader in tobacco control. The state achieved a 38% reduction in adult smoking rates between 1988 and 2006: from 22.7% to 14.0% (2). It now has the second-lowest rate of adult and youth smoking in the nation (3), behind Utah (11.7%) (3), whose largely Mormon population is constrained by a religious prohibition on smoking. Statewide incidence of lung cancer in California has been declining at 4 times the national rate, and, assuming this trend continues, California may be the first state in which lung cancer is no longer the leading cancer cause of death (4). From 1999 to 2004, the CTCP saved the population $86 billion in health care expenditures — a 50-fold return on the $1.8 billion spent on the program during the same period (5). States across the nation and countries from Ireland to New Zealand have adopted strategies pioneered by California to tackle the leading cause of preventable death worldwide (6-8).

We explain the social norm change approach that drives the CTCP and highlight the special role that lawyers have played in this approach to support a wide array of allies in the tobacco control movement. We further describe how lawyers are preparing to play a similar part in the movement to prevent childhood obesity.

## The California Social Norm Change Approach

The goal of its social norm change approach, according to the CTCP, is to indirectly influence "current and potential future tobacco users" by creating an environment in which "tobacco becomes less desirable, less acceptable, and less accessible" (9). The approach presumes that the ideas, values, and behaviors of individuals are moderated by their community. Thus, durable change occurs through shifts in local social norms ranging from unspoken rules of etiquette to the laws on the books. These shifts can occur organically or — as in the case of the CTCP — can result from intentional human intervention. The CTCP has made a deliberate effort to denormalize smoking and other tobacco use in communities across the state.

The Centers for Disease Control and Prevention (CDC) endorsed the California social norm change approach in its 2007 report, *Best Practices for Comprehensive Tobacco Control Programs*, which identifies 5 components of a successful social norm change approach (4):

- State and community interventions, including the adoption of laws, that influence societal organizations, systems, and networks and that encourage individuals to make behavior choices consistent with tobacco-free norms.
- Health communication interventions that "deliver strategic, culturally appropriate, and high-impact messages in sustained . . . campaigns."
- Cessation interventions provided by individual health care providers and quit lines.
- Surveillance and evaluation that monitor "tobacco-related attitudes, behaviors, and health outcomes at regular intervals."
- Substantial funding, sound fiscal management, and robust capacity within a state health department.

CDC recognizes that adopting and implementing state and local laws are essential to a social norm change approach. Laws not only mandate what is allowable but also influence what is socially desirable, acceptable, and accessible. In California, tobacco control laws have been a key factor in the campaign to denormalize smoking and other tobacco use. Proposition 99 decreased smoking rates in California in 2 ways: it deterred consumption through tax-driven price increases on tobacco products, and it earmarked 20% of the state tax for tobacco control programs (10). In the wake of Proposition 99, California passed tobacco control laws (1,11) aimed at limiting secondhand smoke exposure, sales to youth, and tobacco marketing. Moreover, localities throughout California have enacted tobacco control laws that plug loopholes in state laws or cover more ground than do the state laws. For example, state laws prohibit smoking in most enclosed workplaces, in and around playgrounds, and within 20 feet of state buildings. Some municipalities have gone further by enacting laws restricting smoking in outdoor worksites (12), multiunit residences (13), and beaches (14).

### The role of lawyers in the CTCP

Lawyers have played a role in many public health campaigns — from gun control (15) to environmental justice (16) to vehicle, workplace, and product safety (17). The CTCP recognized that legal expertise is vital to sound policy development, so it carved out a special role for lawyers in the California tobacco control movement: providing access to legal resources that support the development of legally viable, enforceable, and defensible state and local laws (18).

In 1997, the CTCP founded the Technical Assistance Legal Center (TALC) as a legal resource for the tobacco control movement in California. TALC does not represent clients, bring lawsuits, or negotiate deals. Instead, TALC provides legal technical assistance to community organizations, local and state health department employees, government attorneys, elected officials and their staff, and others working to denormalize tobacco use through state and local legislation. TALC does not drive the agenda. Rather, it follows the lead of grassroots stakeholders and statewide opinion leaders — identifying and addressing legal issues that arise from their policy goals and their experience developing and implementing these goals.

TALC developed a legal technical assistance model that it has tested and refined for more than a decade. This model breaks down into 5 related parts: conducting legal research and writing, developing model ordinances and policies, creating user-friendly tools, providing training, and offering one-on-one legal technical assistance.

### Legal research and writing

The first part of TALC's model involves conducting in-depth research on the law that applies to potential tobacco control policies. For instance, in the 1990s, a main focus of local tobacco control policy in California was restricting storefront advertising. But the 2001 US Supreme Court case of *Lorillard v Reilly* (19) held that the Federal Cigarette Labeling and Advertising Act and the First Amendment precluded municipalities from adopting and enforcing such advertising restrictions. When the *Lorillard* decision came down, TALC conducted extensive legal research to clarify what was and was not outlawed by the case and to identify viable strategies that remained for limiting tobacco use in California communities (20). TALC sent an e-mail announcement to CTCP stakeholders a few days later explaining that *Lorillard* applies to tobacco advertising regulations but does not affect the ability of communities to regulate how tobacco is sold; thus, cities and counties could still pass ordinances banning the self-service display of tobacco products, requiring a license to sell tobacco, and limiting the location of new tobacco retail outlets. City and county attorneys appreciated TALC's contribution because they generally do not have the time or resources to do this type of research. Instead, they are busy "putting out fires" and keeping abreast of a broad range of legal issues.

### Model ordinances

Second, TALC develops model ordinances for local governments to adopt (and model policies for institutions to adopt) to supplement existing state law and help advocates push the tobacco control agenda forward from the grassroots level. A classic example is TALC's model local tobacco retailer licensing ordinance (21). Tobacco control advocates notified TALC that retailers were disregarding California state laws prohibiting the sale of tobacco products to youth. State and local law enforcement officials were not prioritizing sales-to-youth laws because of budget limitations and competing responsibilities. In response to this problem, TALC developed a model local ordinance requiring every tobacco retailer to obtain a license to sell tobacco. The license can be revoked if the retailer violates state sales-to-youth or other tobacco control laws. To obtain a license, retailers must pay a fee that funds enforcement programs, including youth purchase sting operations. Fifty-three communities in California have adopted a version of this ordinance (22). In a sample of 13 communities, the sales-to-youth rates dropped an average of 68% on adoption and implementation of the ordinance (23).

### User-friendly tools

TALC lawyers realized early on that although government attorneys appreciate the depth and technicality of TALC's model ordinances, nonlawyer stakeholders want concise and accessible print resources that highlight available legal and policy options. The third part of TALC's legal technical assistance model involves creating practical tools — including fact sheets on specific legal topics, checklists representing the key components of TALC model ordinances, how-to memos, and a booklet summarizing the tobacco-related laws that affect California; examples are available at http://talc.phi.org. TALC produces these tools with the assistance of graphic designers so that they are visually appealing and memorable to readers.

### Training

The fourth part of TALC's legal technical assistance model entails offering group training for its model ordinances and tools. TALC hosts its own teleconference and in-person trainings, and TALC attorneys speak at dozens of conferences each year. In a typical TALC presentation, the speaker walks the audience through a given model ordinance and its accompanying tools and shares lessons learned about what has been effective in the field.

### One-on-one technical assistance

Finally, TALC attorneys are available to provide direct technical assistance to stakeholders who write or call. Requests for technical assistance from advocates and others range from basic questions about, for example, the legality of an ordinance banning tobacco billboards in a locality to more in-depth requests for help tailoring a model ordinance to the needs of a given community.

Through TALC's 5-part model of legal technical assistance, public health attorneys have strengthened the capacity of stakeholders in the California tobacco control movement to use the law to create social norm change in their communities.

## The TALC Model Applied to the Childhood Obesity Prevention Movement

In 2007, the Robert Wood Johnson Foundation (RWJF) announced a $500 million commitment to reverse the childhood obesity epidemic by 2015 (24). RWJF was a lead funder of the tobacco control movement for years, and it fully embraces the social norm change approach to public health campaigns (25). In its initial portfolio of grants in the childhood obesity prevention arena, RWJF funded Public Health Law and Policy (PHLP), TALC's parent organization, to launch the National Policy and Legal Analysis Network to Prevent Childhood Obesity (NPLAN). The objective is to develop a national legal technical assistance center for the childhood obesity prevention movement based on the TALC model.

### Challenges identified in the NPLAN needs assessment

To inform the design of NPLAN, PHLP conducted a needs assessment that included more than 100 in-person and telephone interviews and a survey completed by 2,300 stakeholders from sectors and disciplines dealing with nutrition and physical activity (26). The needs assessment elucidated several distinctions between CTCP and the childhood obesity prevention movement that will present fresh challenges for NPLAN.

The tobacco control movement is engaged in a focused campaign against the use of a single product that is addictive and is categorically harmful when used as intended (27). In contrast, childhood obesity prevention advocates confront a complex array of behaviors and products. They have to address both calories consumed and calories burned, and they have to struggle with defining unhealthy foods and activity levels.

The tobacco control movement gained traction from highlighting the negative effects of secondhand smoke on innocent bystanders (28). However, there is nothing that correlates to secondhand smoke in the context of childhood obesity.

The state of the science is another major difference between the 2 movements. Tobacco researchers have generated research findings on nicotine pharmacology, the health effects of tobacco smoke, the economic and marketing tactics of the tobacco industry, and the effectiveness of different policy interventions (29-31). The science around the triggers and consequences of childhood obesity is still in its infancy, posing open questions about which policy interventions will have the greatest effect.

An additional distinction relates to the perception of industry. Whereas tobacco control advocates have almost uniformly viewed industry as the enemy (32), childhood obesity prevention advocates remain divided about whether to work with or against industry.

Finally, TALC provides legal technical assistance statewide to a strongly managed and synchronized campaign. NPLAN will offer legal technical assistance nationally to a movement that has yet to coalesce around an advocacy agenda. Many stakeholders in the childhood obesity prevention arena have to be convinced of the value of legal technical assistance in a social norm change campaign.

### NPLAN design

NPLAN's design takes into account the challenges identified in the needs assessment. Three "learning communities" constitute a core component of the NPLAN organization. Each learning community is made up of research scientists, community and school leaders, and policy experts from around the country, whom NPLAN convenes in monthly conference calls and regular in-person meetings. The learning communities are charged with collaborating on 1 of 3 policy goals: reducing junk food marketing to children, improving food choices and physical activity opportunities in the preschool through high school settings, and enhancing access to healthy food and physical activity in neighborhoods where children live. NPLAN's learning communities serve as a microcosm of the national childhood obesity prevention movement, providing forums to talk about the movement's priorities, opportunities, and constraints. Moreover, the learning communities help NPLAN attorneys anticipate and meet the movement's policy development needs, fostering accountability between NPLAN and the broader constituencies it is set up to serve. By bringing a range of perspectives to the table and creating new alliances, NPLAN's learning communities will drive policy innovation and ensure that all of NPLAN's resource materials are practical, responsive, current, and effective. NPLAN also has appointed an advisory board of national leaders in fields related to childhood obesity prevention to provide strategic guidance and oversight for the new network.

After a year of research and development in conjunction with the learning communities, NPLAN began providing legal technical assistance to the childhood obesity prevention movement in the fall of 2008. NPLAN operates using the basic 5-part model TALC developed in the context of the California tobacco control movement, as exemplified by NPLAN's work on model joint-use agreements. One of the policy priorities that emerged from the needs assessment is to open up school facilities for after-hours recreational use by children and their families, especially in low-income communities that lack safe places to play. NPLAN produced 2 research articles relating to this issue: an in-depth 50-state analysis of tort law that applies to after-hours recreational use of school facilities (aimed at allaying school officials' concerns about liability in the case of injuries) (33) and a 50-state scan of the statutory authority for community use of schools (34). NPLAN also created 4 model joint-use agreements that provide different options for which parties have access to which facilities (35). These models are accompanied by a user-friendly general fact sheet geared toward the public (36) and a checklist of considerations that school and government authorities should make while negotiating joint-use agreements (37). NPLAN presented this material at the 2009 National School Boards Association conference and plans to produce a Web-based seminar that will reach a wider audience. The joint-use products are designed to be stand-alone resources for community leaders and school and government officials, but NPLAN is available to provide one-on-one technical assistance for the products should specific questions arise.

NPLAN is creating similar types of resources to support a range of policy goals. These resources include promoting farmers' markets, community gardens, and healthy mobile vending; requiring fast-food restaurants to post calorie information on menu boards; encouraging schools and other public facilities to have vending contracts for healthy food and beverages; limiting junk food advertising on public school campuses; promoting Complete Streets (which provide safe transportation by ensuring a network of sidewalks, bike lanes, and public transit features); and setting physical activity standards for child-care centers.

It is too early to reach any conclusions about NPLAN's success. NPLAN has engaged an evaluator to conduct evaluations on use and outcomes by using qualitative and quantitative methods to measure indicators of NPLAN's progress toward its goal of empowering stakeholders to support policy innovation and implementation to prevent childhood obesity. Ultimately, the evaluator will provide analytic feedback for the formative development of NPLAN products and services.

## Conclusion

Given the severity of the childhood obesity epidemic in the United States, reversing the epidemic is a challenge that requires a comprehensive social norm change approach. As communities across the nation consider new policies that support healthy eating and physical activity, access to sound, practical legal resources will be essential. By empowering stakeholders to use legal approaches to achieve their aims, NPLAN will be an integral part of RWJF's historic and ambitious effort to reverse the obesity epidemic by 2015.

## Acknowledgments

This article highlights ideas generated at the Symposium on Epidemiologic, Ethical, and Anthropologic Issues in Childhood Overweight and Obesity, sponsored by the Robert Wood Johnson Foundation and the Health Promotion Research Program, a project of the Windward Islands Research and Education Foundation, operated by faculty in the Department of Public Health and Preventive Medicine in the School of Medicine of Saint George's University, Saint George, Grenada.

## References

[1] Cal. Rev. and Tax. Code § 30122(a)(1), § 30123(a), and § 30124(b)(1) (West 2008).

[2] (2006). Adult smoking prevalence.

[3] (2006). Sustaining state programs for tobacco control: data highlights 2006.

[4] (2007). Best practices for comprehensive tobacco control programs — 2007.

[5] Lightwood JM,  Dinno A,  Glantz SA (2008). Effect of the California Tobacco Control Program on personal health care expenditures. PLoS Med.

[6] BBC News Online. All eyes on Ireland's smoking ban.

[7] Laurance J Lancet calls for tobacco ban to save thousands of lives. The Independent (London).

[8] (2006). The facts about smoking and health.

[9] (1998). Model of change: the California experience in tobacco control.

[10] Ross H, Chaloupka FJ (2001). The effect of cigarette prices on youth smoking.

[11] Cal. Lab. Code § 6404.5 (West 2008).

[12] Santa Monica, Cal., Code § 4.44.020 (2004).

[13] Belmont, Cal., Code § 20.5 (2007).

[14] Carmel-by the-Sea, Cal., Code § 8.36.020 (2008).

[15] Dorfman L, Wilbur P, Lingas EO, Woodruff K, Wallack L (2005). Accelerating policy on nutrition: lessons from tobacco, alcohol, firearms, and traffic safety.

[16] Wyenn M, Falender J, Brisson B (2008). Environmental justice: making it a reality. Environmental Law.

[17] Mello MM, Studdert DM, Brennan TA (2006). Obesity — the new frontier of public health law. N Engl J Med.

[18] (2002). Legal technical assistance on tobacco control policy.

[19] 533 U.S. 252 (2001).

[20] (2001). Interpreting the Supreme Court Decision in Massachusetts.

[21] (2008). Model California ordinance requiring a tobacco retailer license (with annotations).

[22] (2008). Matrix of strong local tobacco retailer licensing ordinances in California.

[23] (2007). Tobacco retail licensing is effective.

[24] (2007). Robert Wood Johnson Foundation announces $500-million commitment to reverse childhood obesity in U.S. [press release].

[25] (2008). Childhood obesity.

[26] (2007). Preventing childhood obesity: a five year strategic plan and prospectus for the National Policy and Legal Analysis Network to Prevent Childhood Obesity.

[27] (2004). US Department of Health and Human Services. The health consequences of smoking: a report of the Surgeon General.

[28] (2006). US Department of Health and Human Services. The health consequences of involuntary exposure to tobacco smoke: a report of the Surgeon General.

[29] UCSF Center for Tobacco Control Research and Education.

[30] Global Tobacco Research Network.

[31] Centers for Disease Control and Prevention. Smoking and tobacco use: data and statistics.

[32] (1999). A movement rising: a strategic analysis of US tobacco control advocacy.

[33] Baker T (2009). Liability risks for after-hours use of public school property to reduce obesity: a fifty-state survey.

[34] Rosenbaum S, Lopez N (2009). State laws related to school nutrition and physical education: an analysis of trends and variations and their implications for childhood obesity prevention.

[35] (2009). Model joint use agreements.

[36] (2009). What is a joint use agreement? A fact sheet for parents, students and community members.

[37] (2009). Developing a joint use agreement checklist.

